# Photosynthetic Photon Flux Density Effects on *Portulaca olearacea* in Controlled-Environment Agriculture

**DOI:** 10.3390/plants12203622

**Published:** 2023-10-20

**Authors:** Gediminas Kudirka, Akvilė Viršilė, Kristina Laužikė, Rūta Sutulienė, Giedrė Samuolienė

**Affiliations:** Lithuanian Research Centre for Agriculture and Forestry, Institute of Horticulture, Kauno Str. 30, 54333 Babtai, Lithuania; kristina.lauzike@lammc.lt (K.L.); ruta.sutuliene@lammc.lt (R.S.); giedre.samuoliene@lammc.lt (G.S.)

**Keywords:** controlled-environment agriculture, daily light integral, growth, lighting intensity photosynthesis, purslane

## Abstract

This study aims to evaluate the impacts of the lighting photosynthetic photon flux density (PPFD) on the growth, photosynthesis, and antioxidant response of common purslane (*Portulaca oleracea*) cultivars to determine energy-efficient lighting strategies for CEA. Green and golden purslane cultivars were cultivated in CEA chambers and four experimental treatments consisting of PPFDs of 150, 200, 250, and 300 ± 10 µmol m^−2^s^−1^ were performed, representing daily light integrals (DLIs) of 8.64–17.28 mol m^−2^d^−1^ throughout a 16 h photoperiod. The results show that photoresponses to light PPFDs are cultivar-specific. The green cultivar accumulates 174% more dry weight at 300 PPFD compared to the golden cultivar, and also has a higher LUE, but a lower ETR. Dry weight accumulation, plant height, and leaf area dependence on light intensity do not highlight the economic significance of light PPFD/DLI. The derivative parameter (Δ fresh weight (%)/ΔDLI %) more efficiently explains how the percentage increase in DLI due to an increased PPFD affects the percentage of biomass gain between these PPFD treatments. For both cultivars, the relative fresh weight gain is maximal when the lighting PPFD increases from 200 to 250 µmol m^−2^s^−1^ and declines with PPFD increases from 250 to 300.

## 1. Introduction

Controlled-environment agriculture (CEA) is an advanced agricultural production system (vertical farming, plant factory) with a precisely controlled environment, and it plays an important role in local, seasonless urban vegetable supply. The CEA market size was valued at USD 97.42 million in 2022 and is calculated to reach USD 339.94 million by 2030, rapidly growing at a CAGR of 14.53% from 2023 to 2030 [1]. Leafy vegetables rich in vitamins, minerals, fibers, and antioxidants account for over half of indoor farming operations worldwide [2]. CEA has great potential for improving vegetable productivity, nutritional quality, and food insecurity, but it is energy-intensive [2,3]. Light, temperature, humidity, and plant hydroponic nutrition must be controlled and this is an energy-demanding process. Artificial light is one of the core technologies in CEA and accounts for the largest part of the capital and operation costs [4,5]; therefore, technological and biological strategies for energy-efficient plant lighting in CEA have recently been attracting attention. In the last decade, LED fixture costs have decreased and lighting photon efficacies have increased [5,6]. For example, in the study of Eaton et al., 2023, it was shown that in plant factories, increasing the lighting efficacy from 2.0 μmol J^−1^ to either 2.5 or 3.0 μmol J^−1^ saves 15% and 24% of facility energy consumption, and therefore the energy consumption ranges from 6.2 to 12.0 kWh kg^−1^ fresh weight of lettuce produced depending on the applied technology [7]. However, minimizing energy costs is still critical for CEA profitability [5].

The lighting spectrum, photoperiod, and photosynthetic photon flux density (PPFD) can be optimized, maximizing the efficiency with which plants intercept and use light to produce marketable biomass, which has the potential to reduce input costs and increase yields and profitability [4,5,8]. The light amount (daily light integral, DLI) necessary for optimal lettuce productivity in CEA is acknowledged to be about 14–17 mol m^−2^ per day; in a literature review, a 16 h photoperiod and 250 µmol m^−2^s^−1^ were found to be the average optimum for yield and resource use efficiency in hydroponic lettuce cultivation [9,10]. It was shown that a PPFD ≥ 200 μmol m^−2^s^−1^ results in an improved photosynthetic performance, raises the antioxidant capacity, and improves the nutritional value of lettuce [5,8,10], basil [10,11], rocket [12], kale [13], mizuna [8], and other *Brassicaceae* plants [14]. However, plant responses to the PPFD and its interaction with other cultivation environment conditions are species- and cultivar-specific [10,15]. The response of plant development and growth to environmental conditions, known as phenotypic plasticity, is species-specific; the ability of leaves to intercept light is determined by the leaf area, orientation, and optical properties, as well as their pigment contents [16]. Therefore, the particularity of plant physiological responses emphasizes the need for targeted, individual crop modeling to gain plant-centric insights into productivity and economic parameters [15].

The assortment of vegetables cultivated in CEA is relatively narrow and research is based on high-demand species, such as lettuce, basil, spinach, *Brassicacea* sp., etc. [17]. However, the portfolio of CEA-cultivated species has the potential to be diversified. In recent decades, changes in the human diet and health consciousness have increased society’s interest in healthy diets, resulting in new market trends for healthy foods with high nutritional and organoleptic properties [18]. Wild edible flora may be potential new crops for CEA, serving as a reservoir of diverse micronutrients and nutraceuticals [19,20]. Purslane (*Portulaca olearacea* L.) is a herbaceous annual plant with fleshy stems and thick, succulent leaves. It has received specific attention in recent years due to its exceptional nutritional content, mainly resulting from high contents of omega-3 fatty acids, polysaccharides, alkaloids, terpenoids, sterols, phenols, flavonoids, vitamins A, C, E, and B, and minerals such as potassium, calcium, and magnesium, which provide neuroprotective, antimicrobial, antidiabetic, antioxidant, anti-inflammatory, antiulcerogenic, anticancer, and anticholinesterase properties [18,21]. Known as a common weed, purslane is currently being considered as a future horticultural crop due to its nutritional benefits [22]. Several studies investigated its nutraceutical contents in different plant production systems, including greenhouses and hydroponics [21]. Studies have also investigated the impacts of salinity, nitrogen rates on purslane growth, and nutrient contents [18,23]. It was shown that red and blue light-emitting diodes (LEDs) are superior compared to fluorescent lighting for purslane microgreen growth and nutrient contents [23].

Purslane is a promising candidate for cultivation in CEA; however, it is a C4 halophyte, which undergoes CAM under stress conditions [24]. Therefore, the research knowledge based on lettuce CEA cultivation is not applicable and the individual purslane response to lighting environment conditions should be explored. He and co-authors [24] investigated red/blue 2.2/1 LED light intensity and the lighting duration effects on productivity, photosynthetic light use efficiency, nitrogen metabolism, and the nutritional quality of indoor-grown purslanes. It was concluded that a higher daily light integral (DLI) significantly increased shoot productivity; however, continuous light under the same DLI resulted in reduced growth and a lower light use efficiency. These results encouraged us to explore the lighting PPFD impacts with constant photoperiods, emphasizing the economical efficiency of light usage for purslane productivity. Following this, the objective of our study is to evaluate the impacts of the lighting PPFD on Portulaca oleracea cultivars’ growth, photosynthesis, and antioxidant responses to identify energy-efficient lighting strategies for CEA.

## 2. Results

**Figure 1:**
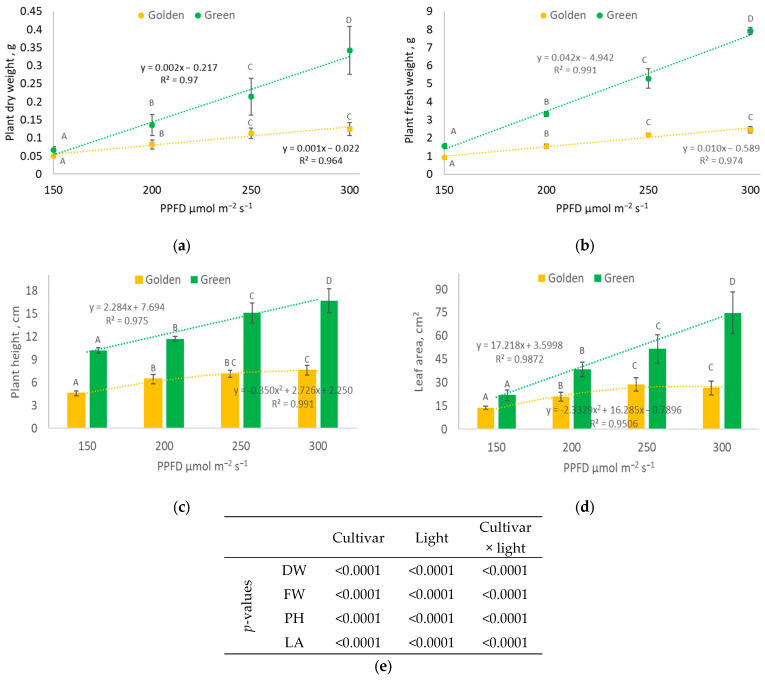
Photosynthetic photon flux density (PPFD) effects on biometric parameters ($\bar{x}\pm SD,n=9)$ of *Portulaca oleracea* green and golden cultivars: (**a**) dry weight (DW), (**b**) fresh weight (FW), (**c**) plant height (PH), (**d**) leaf area (LA). Different letters above the error bars indicate statistically significant differences between means of PPFD treatments within each cultivar according to a one-way ANOVA Tukey’s test, *p* ≤ 0.05. (**e**) Pr > F *p* values indicate the significance of the impact of variables according to a two-way ANOVA.

The selection of optimal crop/cultivar lighting conditions is one of the key factors for the efficiency of plant production in CEA. Lighting intensity directly impacts plant growth parameters (Figure 1), therefore determining plant productivity. Light intensity linearly influences dry and fresh (Figure 1a,b) weights in both purslane cultivars, but the accumulation rate differs. The linear function of the green cultivar dry weight accumulation has a steeper slope (a = 0.002 in the y = ax + b regression equation in Figure 1a) compared with golden cultivar (a = 0.001). However, there is a strong significant correlation between light intensity and dry weight accumulation for both cultivars (R = 0.98 for green and R = 0.98 for golden (Table S1a,b)). The green purslane cultivar accumulates a 34% higher dry weight at 150 µmol m^−2^s^−1^ PPFD exposure and a 180% higher dry weight at 300 µmol m^−2^s^−1^ PPFD compared to the golden cultivar. Dry weight percentages are similar between PPFD treatments (~4.3 for golden and 5.1 for green purslane), as dry and fresh weight accumulation (Figure 1a,b) follow the same trends.

Plant heights and leaf areas (Figure 1c,d) also increase linearly for the green cultivar (R = 0.98 and R = 0.97, respectively; Table S1) but reach a plateau at 250–300 µmol m^−2^s^−1^ PPFD for the golden cultivar, and no statistically significant differences were determined in the measured parameters between these treatments. Notwithstanding, the elevation in the PPFD value from 150 to 200 µmol m^−2^s^−1^ (DLI from 8.64 to 11.52 mol m^−2^ per day) and from 200 to 250 µmol m^−2^s^−1^ (11.52 to 14.4 mol m^−2^ per day) resulted in a remarkable increase in plant leaf area in both cultivars. The leaf area of green purslane plants was determined to be 80 and 34% higher, respectively, while the leaf area increased by 53 and 40% in golden purslane plants. According to the two-way ANOVA results (Figure 1e), both the cultivar and lighting PPFD and their interaction have a significant (*p* < 0.01) impact on the measured biometric parameters.

**Figure 2:**
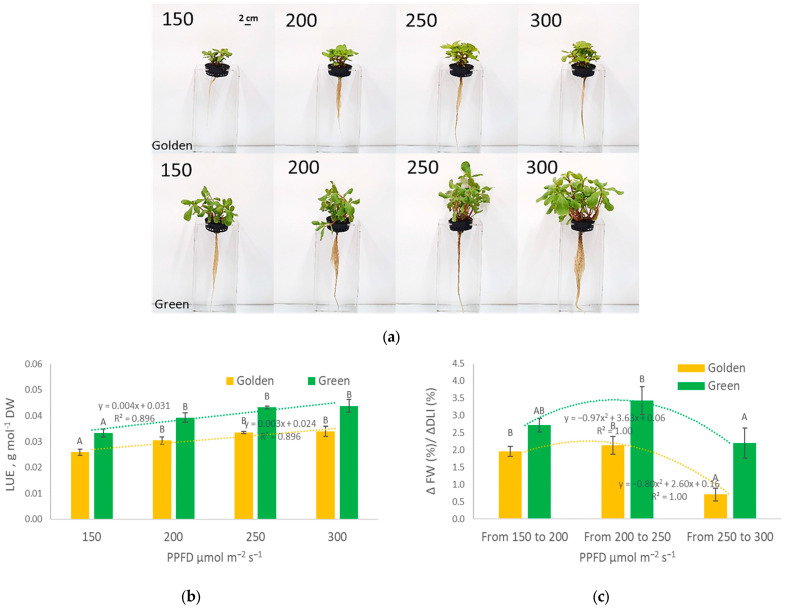
Visual representation (**a**) and the photosynthetic efficiency of *Portulaca olearacea* cultivars: light use efficiency (LUE; (**b**)) and the ratio of the fresh plant weight gain (Δ fresh plant weight (%)) and DLI increment (Δ DLI (%)) due to increased PPFD treatment (**c**) ($\bar{x}\pm SD,\;n=9)$. Different letters above the error bars indicate statistically significant differences between means of treatments within each cultivar according to an ANOVA Tukey’s test, *p* ≤ 0.05.

In practical evaluations, the photosynthetic photon utilization efficiency is not fully reflected in dry and fresh weight accumulation depending on the light intensity. The impact of light intensity increases on *Portulaca olearacea* growth parameters differs at lower and higher PPFD values. Figure 2a–c shows how the increase in DLI due to an increased PPFD (Δ DLI %) between treatments affects the increase in fresh plant weight (Δ fresh weight %). Each 50 µmol m^−2^s^−1^ PPFD increment adds 2.88 mol m^−2^d^−1^ to the DLI compared to the previous treatment; however, the percentage of the total DLI increase declined. The DLI increases by 33, 25, and 20% due to PPFD shifts from 150 to 200, 200 to 250, and 250 to 300 µmol m^−2^s^−1^, respectively. Both cultivars represent the same trend for photon utilization efficiency for fresh weight gain. The relative fresh weight gain is the highest when the PPFD increases from 200 to 250 µmol m^−2^s^−1^ (Δ fresh weight %/Δ DLI % ratio value is 3.83 for green and 2.13 for golden cultivars). Lower Δ fresh weight %/Δ DLI % values when the PPFD increases from 150 to 200 µmol m^−2^s^−1^ indicate that the lighting intensity is still not sufficient for the potential purslane biomass productivity. Decreased ratio values when the PPFD increases from 250 to 300 µmol m^−2^s^−1^ (2.20 for green and 0.71 for golden cultivar) indicate that at this light intensity, additional photosynthetic photons are not efficiently utilized for biomass gain and golden purslane plants possibly experience growth inhibition due to the excess light intensity. While Figure 1a,b shows a linear increase in dry/fresh weight accumulation for green and golden cultivars when the PPFD rises, a growth inhibition is evident at higher lighting intensities considering the parabolic relationship between (Figure 2c) the Δ fresh weight %/Δ DLI % values under different lighting conditions.

**Figure 3:**
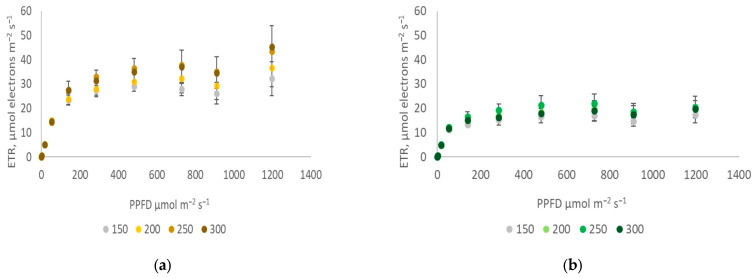
Electron transport rate (ETR) of the golden (**a**) and green (**b**) purslane cultivars ($\bar{x}\pm SD,n=3)$ cultivated under different PPFDs.

During cultivation, the light PPFD has a minor impact on the ETR in purslane compared to the impact of the cultivar (Figure 3a,b). Both the LUE (Figure 2b) and the electron transport rate (ETR; Figure 3) do not differ significantly between cultivars at lower PPFD levels (<200 µmol m^−2^s^−1^). However, at higher PPFDs, the second photosystem’s (PSII) capacity and electron transport rate significantly differ between both purslane cultivars. The golden cultivar (Figure 3a) in the plateau phase has a 63% higher ETR compared to the green cultivar (Figure 3b), while at 250–300 µmol m^−2^s^−1^, the green cultivar has a 1.4–2.1 times higher LUE compared to the golden cultivar. This corresponds to morphological features—a higher leaf area (Figure 1c), chlorophyll index, and nitrogen balance index (NBI) (Table 1) in the green cultivar—that enable them to efficiently capture and utilize light energy at the applied CO_2_ level.

The NBI index decreases as the flavonoid index increases at higher light intensities, and this trend is common for both cultivars (Table 1); the NBI was determined to be 29% and 38% lower at 300 compared to 150 µmol m^−2^s^−1^ PPFD in green and golden purslane cultivars, respectively. The cultivar and applied light intensity have a significant impact on all measured optical indices, while their interaction has a statistically significant impact on only the chlorophyll index. In the golden cultivar, a reduced chlorophyll index at 250 and 300 µmol m^−2^s^−1^ indicates possible photostress conditions, and also corresponds with a lower DPPH free radical scavenging activity at these PPFD levels (Table 2). Taken as a whole, though the measured antioxidant indices are comparable between the two purslane cultivars, the antioxidant response to different PPFDs is cultivar-specific and depends on light intensity and their interaction. At 150–200 µmol m^−2^s^−1^ DPPH, the free radical scavenging activity is similar for both cultivars (according to a Student’s t-test). However, for the green cultivar, a further increase in the PPFD strongly (R > 0.8) correlates with DPPH, FRAP, and total phenolic compound (TPC) contents, as well as biometric indices (Table S1a), indicating a positive plant reaction to higher cultivation light PPFDs. The DPPH free radical scavenging activity and the FRAP antioxidant power were determined to be 30% higher at 300 compared to 150 µmol m^−2^s^−1^. Meanwhile, in golden purslane, 250 µmol m^−2^s^−1^ light treatment resulted in the lowest measured antioxidant parameters, and a strong significant correlation was observed only between TPC and DPPH (R = 0.81) and FRAP (R = 0.93) values (Table S1b).

**Figure 4:**
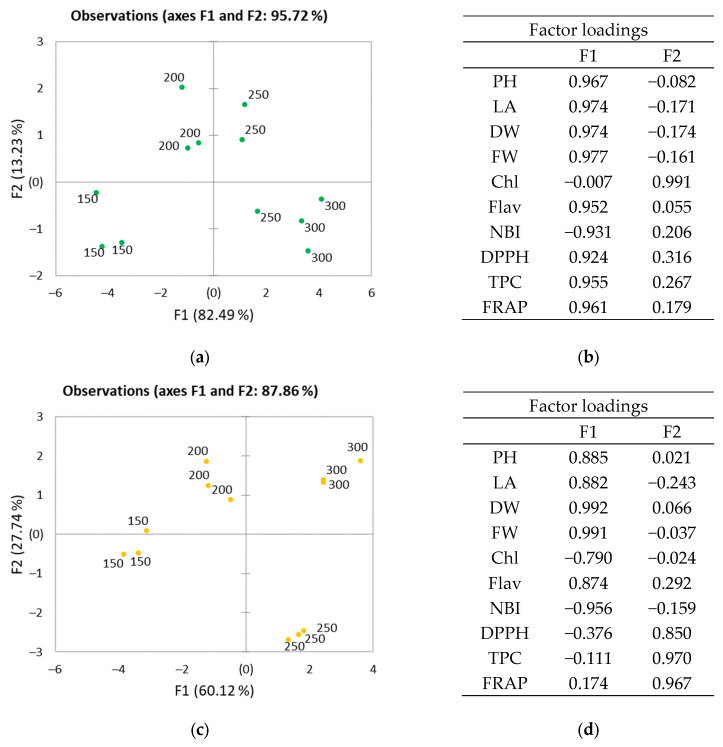
Principal component analysis (PCA) scatter plot (**a**,**c**) (n = 3 experimental repetitions) and factor loadings (**b**,**d**) of *Portulaca oleracea* cultivated under different light intensities: (**a**,**b**) green cultivar; (**c**,**d**) golden cultivar.

The PCA score scatterplot (Figure 4a,c) shows the average coordinates of all measured parameters in the two purslane cultivars under different light intensities. The first two components, F1 and F2, correspond to 95.72% and 87.86% of the total variation for green and golden purslane cultivars, respectively. The majority of variation between variables for the green cultivar is explained by the F1 (82.49%) component, which, according to factor loadings (Figure 4b,d), is strongly related to biometric parameters (leaf area, plant height, dry and fresh weight) and antioxidant parameters. In the golden cultivar, the variation according to the F1 component is influenced by biometric but not antioxidant parameters. For both cultivars, no significant differences were observed between 250 and 300 µmol m^−2^s^−1^ PPFD treatments according the F1 component. According to the F2 component, for the green cultivar, 250 and 300 µmol m^−2^s^−1^ PPFD treatments significantly differ according to the chlorophyll index, while for the golden cultivar they differ according to the DPPH free radical scavenging activity, FRAP antioxidant power, and total phenolic content.

## 3. Discussion

Providing an adequate lighting PPFD in CEA is expensive, and electricity costs could limit the widespread adoption of indoor plant cultivation systems on a commercial scale [10]. Therefore, optimal lighting conditions and cultivars with a higher photosynthetic efficiency should be selected for sustainable crop production. Insights from plant photoresponses to different PPFD levels can be utilized to optimize energy efficiency [3,25]. For purslane, similar to lettuce [16], the light intensity linearly influences dry weight in both cultivars, but the photosynthetic efficiency differs, as indicated by the steeper slope of dependence between PPFD and dry weight for the green cultivar compared to the golden cultivar. The green cultivar accumulates 174% more dry weight at 300 PPFD compared to the golden cultivar. It also has a higher LUE, but a lower ETR, compared to the golden cultivar. This corresponds to the morphological features of the green cultivar —a higher leaf area (Figure 1d), chlorophyll index, and nitrogen balance index (NBI) (Table 1)—which enable it to efficiently capture and utilize light energy under particular cultivation conditions. Tarr et al. (2023) [15] explained that the leaf photosynthetic rate increases linearly with the PPFD until the light saturation point, after which a greater PPFD does not further increase photosynthesis. Increasing the PPFD above the light saturation point can reduce the light use efficiency [26,27], as energy inputs increase without proportional yield responses. The differences in the purslane cultivars’ responses to light PPFDs might be attributed to their phenotypes, as they have different leaf pigmentations and morphologies. A high chlorophyll content in green cultivars may potentially increase the ETR, CO_2_ assimilation rate, and growth rate. Kim and van Iersel (2022) also proposed that the anthocyanin content in leaves may be associated with reduced growth, because light absorption by anthocyanins reduces the amount of photons available for photosynthesis [28]. Screening for rapidly growing crops and increasing the efficiency of vertical farms, Jayalath and van Iersel (2021) [29] discovered that plants with a greater projected canopy size, and therefore a greater total incident light over the growing period, had faster growth rates. In our study, the different canopy morphologies of the cultivars could also explain the contrasting productivity of green and golden purslane. Accordingly, the positive correlation between green purslane productivity and antioxidant indices, which was not observed in golden purslane, confirms the different physiological mechanisms of the two cultivars, and this could be related to a lower PSII capacity in green purslane.

Dry weight accumulation, plant height, and leaf area dependence on light intensity do not highlight the economic significance of light PPFDs/DLIs. In this study, a higher PPFD corresponded to increased DLI values, as the photoperiod was kept constant. The derivative parameter (Δ fresh weight (%)/ΔDLI %) explains how the percentage increase in DLI due to an increased PPFD affects the percentage of biomass gain between these PPFD treatments. Relative values are qualitative justification of the most efficient light intensity. The same tendency is observed for both cultivars, however, it is more clear for the golden cultivar. The relative fresh weight gain is maximal when lighting PPFD is increased from 200 to 250 µmol m^−2^s^−1^ (DLI increase from 11.52 to 14.4 moles m^−2^ per day) and declines with a PPFD increase from 250 to 300 µmol m^−2^s^−1^ (DLI increase from 14.4 to 17.28 mol m^−2^ per day). This suggests that the most efficient light energy conversion to purslane biomass occurs at 250 µmol m^−2^s^−1^. When the light saturation point is reached, only increasing the photoperiod would promote further efficient biomass gains [5,8,30]. Higher light intensities are reported [30] to disturb the balance between energy supply and energy consumption in plants; the amount of energy absorbed for photosynthesis continually overcomes the metabolic energy needed, which results in the accumulation of excess energy in thylakoid membranes which has the potential to harm PSII and cause photo-inhibition [30]. He et al. (2023) [24] investigated different PPFD and photoperiod combinations for purslane growth and photosynthesis and concluded that the optimal productivity was obtained at 18 h at 320 µmol m^−2^s^−1^ compared to continuous lighting or higher PPFDs with shorter photoperiod. In addition, higher PPFDs resulted in higher mineral and nutrient contents [24,31].

The results of this study indicate that for reasonable economic efficiency of purslane lighting in CEA, the direct evaluation of plant photoresponses to the light PPFD is not sufficient. The derivative parameter (Δ fresh weight (%)/ΔDLI %) better represents the utilization of electric energy at different PPFD levels for purslane biomass gain. However, the cultivar-specific response to the applied PPFD highlights the necessity for more specific research on efficient CEA technologies.

## 4. Materials and Methods

**Figure 5:**
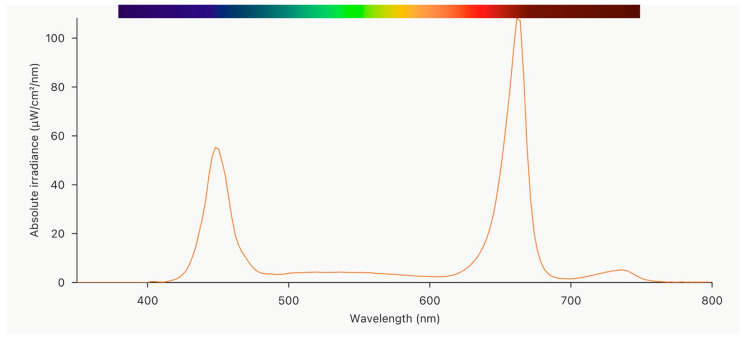
Light spectral distribution (measured at 250 µmol m^−2^s^−1^).

Plant cultivation. Experiments were performed in a walk-in, controlled-environment chamber, replicating common vertical farming conditions. Day/night temperatures of 21/17 ± 2 °C were established within a 16 h photoperiod, with a relative humidity of 50–60% and a CO_2_ level of 1000 ppm within the chamber. Artificial lighting was provided by 4-channel controllable light emitting diode (LED) units (TUAS GTR 2V 0021096109 C1 DL ST, Tungsram, Hungary) with an equal spectral composition ((Figure 5) deep red 61%, blue 20%, white 15%, far red 4%) in all lighting treatments from sowing. The choice of the lighting spectrum was based on previous knowledge on light spectrum impacts on leafy vegetables [32] and adapted according to the lighting unit options. A blue, red, and far red light combination was shown to be efficient for different plants, including purslane microgreens [23,32], while white light was added to make the cultivation light more appropriate for human vision and plant inspections [33].

Seeds of green and golden cultivars of common purslane (*Portulaca oleracea* L.) (CN seeds Pymoor, Ely, Cambridgeshire, UK) were germinated in rockwool cubes (20 × 20 mm; Grodan, Roermond, The Netherlands) and soaked in distilled water under a lighting photosynthetic photon flux density (PPFD) of 200 µmol m^−2^s^−1^. On the 10th day after germination, seedlings were thinned out, leaving 5 plants per cube, and transferred into deep water culture (DWC) hydroponic systems. Each experimental PPFD treatment consisted of 3 (each tank represents experimental replication) DWC tanks of 40 L volume, with 12 tanks in total. Each DWC tank contained 12 mesh pots, with 7 plants in each. A commercial hydroponic nutrient solution was used (Hydro A (NPK(3-0-1), CaO 5.9%, MgO 0.4%) and Hydro B (NPK 1-3-6, MgO 1.4%); Plagron Ommelpad, NL), using 2.5 mL of each component per liter of water. The initial buffer capacity of the hydroponic solution was enhanced by the addition of 3 mM MES (2-(N-morpholino) ethane sulfonic acid) as a buffering agent and therefore pH values were passively maintained in the range of 6.0–6.5 throughout the cultivation period.

**Figure 6:**
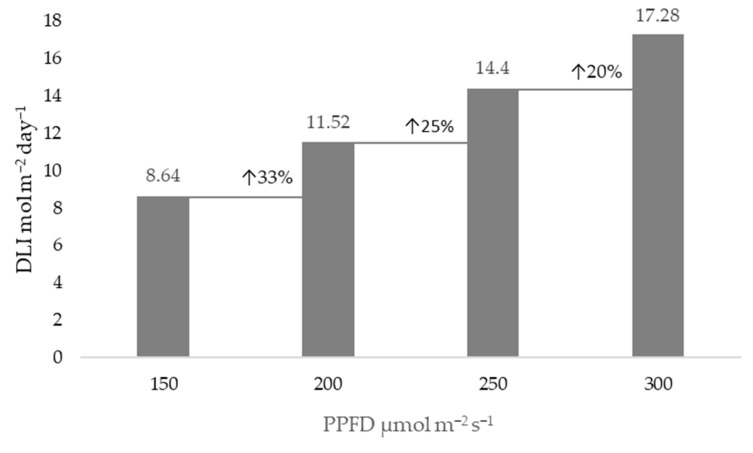
Experimental setup and daily light integral (DLI) differences (%) between PPFD treatments. Numbers next to the arrows represent the percentage of the DLI increase between different PPFD treatments.

Lighting PPFD treatments. Photosynthetic photon flux densities (PPFDs) common in controlled-environment agriculture were selected for the experimental treatments. Four experimental treatments of PPFDs of 150, 200, 250, and 300 ± 10 µmol m^−2^s^−1^ were performed, representing daily light integrals (DLIs) of 8.64–17.28 mol m^−2^d^−1^ throughout the 16 h photoperiod (Figure 6), while maintaining equal proportions of the spectral composition. The PPFD was periodically measured and regulated at the plant level using a photometer–radiometer (RF-100, Sonopan, Poland).

Analyses were performed 35 days after sowing. All measurements in each experimental replication were performed in 3 analytical replications (9 measurements per PPFD treatment in total). Plant material was lyophilized (FD-7, SIA Cryogenic and Vacuum Systems, Latvia) and ground, and dry plant material was used for the determination of antioxidant properties.

Biometric measurements. Plant above-ground parts were used to determine plant height (PH) and fresh (FW) and dry (DW; after lyophilization) weights. The leaf area (LA) was determined using a benchtop leaf area meter (AT Delta–T Devices, Cambridge, UK). To calculate the LUE (light use efficiency), the shoot dry weight was divided by the total incident light that the plant received over the growing period. The ratio of Δ fresh plant weight (%)/Δ DLI (%) was calculated to compare the impact of light intensity increases under different PPFD treatments.

The ETR (electron transport rate) was determined using an imaging-PAM fluorometer (M-Series MAXI-Version (Walz, Effeltrich, Germany)) on light-acclimated (30 min) plants. Three plants from each experimental replication (9 plants per PPFD treatment) were used for measurements. PPFDs were gradually increased at fixed time intervals from 0, 2, 18, 54, 140, 285, 481, 728, and 910 to 1196 µmol s^−1^m^−2^ and chlorophyll fluorescence parameters were recorded.

Leaf optical indexes. A Dualex meter (Force-A, France) was used to determine chlorophyll (Chl, µg cm^−2^) and flavonol (Flav, relative absorbance units) contents and nitrogen balance indexes (NBIs, relative units).

Antioxidant activity and total phenolic content determination. Antioxidant activity was expressed as DPPH (2-diphenyl-1-picrylhydrazyl) free radical scavenging activity and ferric reduction antioxidant power (FRAP), and the total contents of phenolic compounds (TPCs) were evaluated in dry plant material. Lyophilized plant material (0.01 g) was ground with 5 mL 80% methanol, incubated for 24 h, and centrifuged (10 min, 4500 rpm; Z366, Hermle, Germany), and the supernatant was used for analysis.

A stable 126.8 µM DPPH (100% purity; Sigma-Aldrich, Burlington, MA, USA) solution was prepared in methanol. Analyses were conducted in 96-well plates by mixing 290 µL of DPPH solution with 20 µL of plant extract in each well; the absorbance was read at 515 nm (Spectro-star Nano, BMG Labtech microplate reader, Germany) at the 16th minute [34]. Results were expressed as µmol of DPPH scavenged per 1 g of dry plant weight (µmol g^−1^ DW).

The FRAP method is based on Fe^3+^ ion reduction to Fe^2+^. A fresh working solution was prepared by mixing the following solutions in a 10:1:1 volumetric ratio: 10 mM TPTZ (2,4,6-tripyridyl-s-triazine) solution in 300 mM, pH 3.6 acetate buffer, 40 mM HCl, and 20 mM FeCl_3_ × 6H_2_O. Analyses were conducted in 96-well plates by mixing 290 µL of working solution with 20 µL of plant extract in each well and incubating it in the dark for 30 min; the absorbance was read at 593 nm (Spectro-star Nano microplate reader, BMG Labtech, Ortenberg, Germany) [35]. Solutions of Fe_2_(SO_4_)_3_ (0.005–0.5 mM) (Iron (III) sulphate; 97% purity; Sigma-Aldrich, Burlington, MA, USA) were used to determine the calibration curve. Results were expressed as µmol of Fe^2+^ reduced by g^−1^ of dry plant weight (µmol g^−1^ DW).

Analyses of total phenolic compound (TPC) contents were conducted by mixing 20 µL of plant extract with 20 µL of 10% (*w*/*v*) Folin–Ciocalteu reagent and 160 µL of 1 M Na_2_CO_3_ solution and incubating it in the dark for 20 min. Afterwards, absorbance was read at 765 nm (Spectro-star Nano, BMG Labtech microplate reader, Germany) [36]. The contents of total phenolic compounds were quantified as gallic acid equivalents according to the calibration curve. Results were expressed by mg of TPC per dry plant weight (mg g^−1^ DW).

Statistical analysis. The results are presented as the average ± standard deviation of 3 experimental and 3 analytical replications, n = 9 per PPFD treatment. A one-way ANOVA, using Tukey’s HSD test, was employed to determine differences between light treatments; a two-way ANOVA was used to evaluate the significance of the impact and interaction of variables (cultivar and lighting treatment) at the confidence level of *p* ≤ 0.05. For result modeling, a correlation analysis and a multivariate principal component analysis (PCA) were performed. Data were evaluated using Microsoft^®^ Excel^®^ for Microsoft 365 MSO (Version 2309 Build 16.0.16827.20166) 64–bit and compatible XLStat version 2022.1.2.1279 (64–bit) (Addinsoft, Paris, France) software packages.

## 5. Conclusions

For a reasonable economic efficiency of plant lighting in CEA, the direct evaluation of plant photoresponses to light PPFDs/DLIs is not sufficient. For both green and golden purslane cultivars, increasing the light intensity linearly influences dry and fresh weight accumulation; however, the derivative parameter (Δ fresh weight (%)/ΔDLI %) shows that biomass gains, considering the lighting DLI input, decline when the PPFD increases from 250 to 300 µmol m^−2^s^−1^ (DLI increases from 14.4 to 17.28 mol m^−2^ per day), and the most efficient light energy conversion to purslane biomass occurs at 250 µmol m^−2^s^−1^. However, the species- and even cultivar-specific responses to the applied PPFD highlight the necessity for more specific research on efficient CEA technologies.

## Figures and Tables

**Table 1:** Leaf optical indices in green and golden purslane cultivars ($\bar{x}\pm SD,\;n=9)$. Different letters within the column indicate statistically significant differences between means within each cultivar according to a one-way ANOVA Tukey’s test at the confidence level *p* ≤ 0.05. Pr > F *p* values indicate the significance of the impact of the variable according to a two-way ANOVA. Chl—chlorophyll content (µg cm^−2^), Flav—flavonol content (relative absorbance units), NBI—nitrogen balance index (relative units).

Cultivar	PPFD, µmol m^−2^s^−1^	Leaf Optical Indexes		
		Chl	Flav	NBI
Green	150	23.39 ± 1.57 a	0.64 ± 0.10 a	37.44 ± 5.24 c
	200	25.52 ± 1.48 b	0.78 ± 0.11 b	33.51 ± 4.99 b
	250	25.05 ± 1.83 b	0.81 ± 0.11 b	31.42 ± 4.54 b
	300	23.40 ± 1.69 a	0.90 ± 0.14 c	26.53 ± 4.72 a
Golden	150	20.38 ± 3.11 b	0.73 ± 0.09 a	27.82 ± 3.49 d
	200	20.53 ± 2.28 b	0.88 ± 0.09 b	23.58 ± 3.18 c
	250	18.01 ± 1.66 a	0.89 ± 0.08 b	20.58 ± 3.02 b
	300	16.53 ± 3.45 a	0.97 ± 0.10 c	17.27 ± 3.81 a
*p*-values, Pr > F				
Cultivar		<0.0001	<0.0001	<0.0001
Light intensity		<0.0001	<0.0001	<0.0001
Cultivar × Light intensity		0.043	0.805	0.925

**Table 2:** Cultivar-dependant antioxidant activity and total phenolic compound concentration distribution ($\bar{x}\pm SD,\;n=5)$. Different letters within the column indicate statistically significant differences between means of cultivar according to a one-way ANOVA Tukey’s test at a confidence level of *p* ≤ 0.05. Pr > F *p* values indicate the significance of the impact of the variable according to a two-way ANOVA. DW—dry plant weight.

Cultivar	PPFD, µmol m^−2^s^−1^	DPPH, mmol g^−1^ DW	FRAP, µmol g^−1^ DW	TPC, mg g^−1^ DW
Green	150	108.86 ± 0.55 a	162.08 ± 3.74 a	8.32 ± 0.05 a
	200	132.64 ± 3.81 b	188.77 ± 4.64 b	10.11 ± 0.13 b
	250	138.28 ± 5.50 bc	208.47 ± 5.79 bc	10.60 ± 0.22 c
	300	144.54 ± 0.94 c	213.27 ± 1.31 c	11.20 ± 0.09 c
Golden	150	107.69 ± 9.56 b	141.47 ± 0.60 b	7.49 ± 0.05 b
	200	125.76 ± 4.36 c	151.78 ± 3.41 c	7.94 ± 0.06 c
	250	80.61 ± 0.85 a	128.18 ± 3.34 a	6.15 ± 0.14 a
	300	106.59 ± 3.71 b	159.65 ± 2.49 c	8.12 ± 0.08 c
*p*-values, Pr > F				
Cultivar		<0.0001	<0.0001	<0.0001
Light intensity		<0.0001	<0.0001	<0.0001
Cultivar × Light intensity		<0.0001	<0.0001	<0.0001

## Data Availability

Not applicable.

## Supplementary Materials

The following supporting information can be downloaded at: https://www.mdpi.com/article/10.3390/plants12203622/s1, Table S1: Pearson correlation matrix of parameters, measured in *Portulaca olearacea*.
