# Supplementary material for: Photosynthetic Photon Flux Density Effects on Portulaca olearacea in Controlled-Environment Agriculture
Source: Plants (Basel). 2023 Oct 20;12(20):3622. doi: 10.3390/plants12203622 (PMC10609831; doi:10.3390/plants12203622)
Supplement: Supplementary file 1 [file plants-12-03622-s001.zip › plants-2646426-supplementary.pdf]

plants

Supplement 1a. Pearson correlation matrix of parameters, measured in green P*ortulaca olearacea*. Determination coefficient values in bold are statistically significant when $p \leq 0.05$.

Variables	PPFD	Height	Leaf area	Dry weight	Fresh weight	Chl	Flav	NBI	DPPH	TPC	FRAP
PPFD	**1**	**0,977**	**0,973**	**0,983**	**0,988**	-0,039	**0,917**	**-0,901**	**0,905**	**0,941**	**0,947**
Height	**0,977**	**1**	**0,956**	**0,984**	**0,986**	-0,077	**0,874**	**-0,858**	**0,856**	**0,890**	**0,916**
Leaf area	**0,973**	**0,956**	**1**	**0,979**	**0,983**	-0,168	**0,923**	**-0,932**	**0,833**	**0,888**	**0,892**
Dry weight	**0,983**	**0,984**	**0,979**	**1**	**0,998**	-0,174	**0,891**	**-0,905**	**0,840**	**0,878**	**0,912**
Fresh weight	**0,988**	**0,986**	**0,983**	**0,998**	**1**	-0,159	**0,899**	**-0,907**	**0,845**	**0,886**	**0,907**
Chl	-0,039	-0,077	-0,168	-0,174	-0,159	**1**	0,066	0,209	0,281	0,246	0,158
Flav	**0,917**	**0,874**	**0,923**	**0,891**	**0,899**	0,066	**1**	**-0,949**	**0,871**	**0,911**	**0,890**
NBI	**-0,901**	**-0,858**	**-0,932**	**-0,905**	**-0,907**	0,209	**-0,949**	**1**	**-0,789**	**-0,831**	**-0,845**
DPPH	**0,905**	**0,856**	**0,833**	**0,840**	**0,845**	0,281	**0,871**	**-0,789**	**1**	**0,984**	**0,951**
TPC	**0,941**	**0,890**	**0,888**	**0,878**	**0,886**	0,246	**0,911**	**-0,831**	**0,984**	**1**	**0,967**
FRAP	**0,947**	**0,916**	**0,892**	**0,912**	**0,907**	0,158	**0,890**	**-0,845**	**0,951**	**0,967**	**1**

Supplement 1b. Pearson correlation matrix of parameters, measured in golden *Portulaca olearacea*. Determination coefficient values in bold are statistically significant, when $p \leq 0.05$.

Variables	PPFD	Height	Leaf area	Dry weight	Fresh weight	Chl	Flav	NBI	DPPH	TPC	FRAP
PPFD	**1**	**0,884**	**0,822**	**0,989**	**0,979**	**-0,753**	**0,877**	**-0,931**	-0,318	0,014	0,286
Height	**0,884**	**1**	**0,810**	**0,864**	**0,878**	-0,502	**0,811**	**-0,777**	-0,289	-0,071	0,156
Leaf area	**0,822**	**0,810**	**1**	**0,849**	**0,909**	**-0,587**	**0,672**	**-0,748**	-0,463	-0,337	-0,082
Dry weight	**0,989**	**0,864**	**0,849**	**1**	**0,989**	**-0,789**	**0,881**	**-0,951**	-0,350	-0,029	0,247
Fresh weight	**0,979**	**0,878**	**0,909**	**0,989**	**1**	**-0,767**	**0,833**	**-0,919**	-0,416	-0,129	0,147
Chl	**-0,753**	-0,502	**-0,587**	**-0,789**	**-0,767**	**1**	-0,553	**0,873**	0,271	0,074	-0,158
Flav	**0,877**	**0,811**	**0,672**	**0,881**	**0,833**	-0,553	**1**	**-0,881**	-0,100	0,166	0,422
NBI	**-0,931**	**-0,777**	**-0,748**	**-0,951**	**-0,919**	**0,873**	**-0,881**	**1**	0,203	-0,020	-0,304
DPPH	-0,318	-0,289	-0,463	-0,350	-0,416	0,271	-0,100	0,203	**1**	**0,812**	**0,713**
TPC	0,014	-0,071	-0,337	-0,029	-0,129	0,074	0,166	-0,020	**0,812**	**1**	**0,928**
FRAP	0,286	0,156	-0,082	0,247	0,147	-0,158	0,422	-0,304	**0,713**	**0,928**	**1**
